# One-third of an Archivial Series of Papillary Thyroid Cancer (Years 2007–2015) Has Coexistent Chronic Lymphocytic Thyroiditis, Which Is Associated with a More Favorable Tumor-Node-Metastasis Staging

**DOI:** 10.3389/fendo.2017.00337

**Published:** 2017-12-01

**Authors:** Antonio Ieni, Roberto Vita, Emilia Magliolo, Mariacarmela Santarpia, Flavia Di Bari, Salvatore Benvenga, Giovanni Tuccari

**Affiliations:** ^1^Department of Human Pathology of Adult and Evolutive Age “Gaetano Barresi”—Section of Pathological Anatomy, University of Messina, Messina, Italy; ^2^Department of Clinical and Experimental Medicine, University of Messina, Messina, Italy; ^3^Pathology Unit, Taormina Hospital, Messina, Italy; ^4^Department of Human Pathology of Adult and Evolutive Age “Gaetano Barresi”—Section of Oncology, University of Messina, Messina, Italy; ^5^Master Program on Childhood, Adolescent and Women’s Endocrine Health, University of Messina, Messina, Italy; ^6^Interdepartmental Program of Molecular & Clinical Endocrinology and Women’s Endocrine Health, University Hospital, Azienda Ospedaliera Universitaria Policlinico G. Martino, Messina, Italy

**Keywords:** chronic lymphocytic thyroiditis, autoimmune thyroiditis, papillary thyroid cancer, autoimmunity, cancer

## Abstract

The significance and impact of the coexistence of chronic lymphocytic thyroiditis (CLT) with thyroid cancer is still debated. To verify the influence of CLT on papillary thyroid cancer (PTC), we retrospectively collected 505 PTC cases and analyzed age at diagnosis, sex, size, lymph node status, and staging. We found that CLT was present in 168 PTC (33.3%). Compared with the 337 patients without CLT (non-CLT), CLT patients were younger (44.42 ± 13.72 vs. 47.21 ± 13.76 years, *P* = 0.03), had smaller tumors (9.39 ± 6.10 vs. 12 ± 9.71 mm, *P* = 0.002), and lower rate of lymph node metastases (12.5 vs. 21.96%, *P* = 0.01, OR = 0.508). Tumor-node-metastasis (TNM) staging (T1a through T4) was more favorable for the CLT group compared to the non-CLT group (for instance, T1a = 65.5 vs. 49.8%, T3 = 4.8 vs. 23.4%). This study shows that one in three patients with PTC harbors CLT, which is associated with a more favorable TNM staging, consistently with a favorable outlook of PTC.

## Introduction

Autoimmune thyroid diseases represent the most frequent autoimmune disorders in humans (1). Among the spectrum of autoimmune thyroid diseases, chronic lymphocytic thyroiditis (CLT)/Hashimoto’s thyroiditis (CLT) is the leading one in terms of prevalence and incidence (1, 2). At diagnosis, CLT may present with an enlarged thyroid (goiter), a normally sized thyroid, or with an hypotrophic/atrophic thyroid (1, 3, 4). As highlighted in the first description by Dr. Hakaru Hashimoto, CLT is characterized by an intense lymphoid infiltration, which can organize into follicles and germinal centers. Additional features include the presence of Hürthle cells, which result from thyrocytes metaplasia, atrophy, and interstitial fibrosis (5).

It is well known that CLT may coexist with thyroid cancer particularly with papillary thyroid cancer (PTC), but the significance of this coexistence still remains controversial. In fact, several papers have reported an increased risk of developing PTC in patients with preexisting CLT (6–10). Some authors have suggested that the coexistence of PTC and CLT is associated with better prognosis, with a lower rate of lymph node and distant metastasis, and lower rate of recurrence (9–13).

Based on previous reports (14, 15) highlighting an increase in the incidence of PTC and CLT cases in Sicily, which is the southernmost region of Italy, we performed a retrospective study to determine the frequency and the influence of CLT on PTC.

## Materials and Methods

We retrospectively reviewed the electronic records of the 8-year period 2007–2015 in the Pathology Units of the University Hospital of Messina and the Taormina Hospital, which are located within two municipalities in northeastern Sicily, Italy. Queries were run using the keywords “thyroid,” “papillary carcinoma,” “Hashimoto,” and “chronic lymphocytic thyroiditis.” Therefore, other types of thyroid malignancy (follicular carcinomas, anaplastic carcinomas, etc.) were disregarded. Stained sections of all paraffin-embedded PTC specimens retrieved in order to confirm the previous diagnosis and association between PTC and HT were reviewed. The pathologists considered as “Hashimoto’s thyroiditis” those cases showing histological evidence of lymphocytic infiltration with germinal center formation and Hürthle cell metaplasia. The presence of a less marked, isolated lymphocytic infiltration was not considered indicative of Hashimoto’s thyroiditis, and was labeled as “non-specific chronic thyroiditis.”

For all cases, the following data were available: age at diagnosis, sex, tumor size, histotype, lymph node status, and pathological staging. All cases were staged using the tumor-node-metastasis (TNM) criteria of the American Joint Committee on Cancer/Union International Contre le Cancer staging for thyroid cancer (16).

This was a retrospective study that did not involve interventions in human subjects.

Categorical variables were handled with the χ^2^-test or the Fisher’s exact test as appropriate. Odds ratio (OR) and 95% confidence interval (95% CI) are given. Comparison between continuous variables of two or more groups was performed by *t*-test for unpaired data or ANOVA. *P*-values less than 0.05 were considered statistically significant, while *P* values between 0.10 and 0.05 were considered borderline significant.

## Results

**Figure 1:**
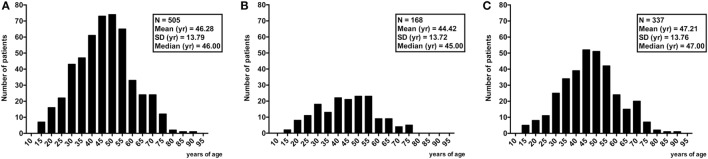
Age distribution of all 505 patients with papillary thyroid cancer **(A)** and the two groups with [*n* = 168 **(B)**] or without [*n* = 337 **(C)**] chronic lymphocytic thyroiditis.

A total of 505 consecutive PTC cases were retrieved and confirmed to be such. Gender distribution was 399 (79%) females and 106 (21%) males. Mean age of 46.29 years (range 14–91) (Figure 1; Table 1).

**Table 1:** Demographics and characteristics of the 505 consecutive papillary thyroid cancers retrospectively reviewed.

Parameters	Patients (*n* = 505)
Sex	399 (79%) F 106 (21%) M
Age (m ± SD)	46.28 ± 13.79
Size (mm)	11.13 ± 8.76
Chronic lymphocytic thyroidits	337 (66.74%) absence 168 (33.26%) presence
Histotype	256 (50.69%) classic variant 183 (36.24%) follicular variant 39 (7.72%) sclerosing 9 (1.78%) tall cell 9 (1.78%) Warthin-like 8 (1.58%) hobnail/micropapillary 1 (0.19%) cribriform
Lymph node	410 (81.18%) N0 95 (18.82%) N+
pT	278 (55.04%) T1a 105 (20.79%) T1b 34 (6.74%) T2 87 (17.23%) T3 1 (0.20%) T4

The mean nodule size was 11.13 mm (range 1–80 mm). Of the 505 PTC cases, the classic variant and the cribriform variant were the commonest (50.7%) and the rarest (0.2%), respectively. Concerning lymph node status, 410 cases (81.18%) were N0, whereas the remaining 95 (18.82%) were N+. Half of PTC cases were staged T1a (55.0%), one-fifth T1b (20.8%), one-fifteenth as T2 (6.74%), one-sixth as T3 (17.23%), and one-fifty hundredths as T4 (0.20%). All data are summarized in Table 1.

Based on whether or not histological signs of thyroiditis were detected, two groups of patients were formed: the CLT group [*n* = 168 (33.3%)], and the non-CLT group [*n* = 337 (66.7%)]. Table 2 contrasts the analyzed characteristics in the two groups. CLT was more common in females than in males [146/399 (36.6%) vs. 22/106 (20.7%), *P* = 0.002, OR = 2.20, 95% CI = 1.32–3.67]. Based on sex, there was no difference between histotypes frequency (*P* = 0.489; not shown in Table 2).

**Table 2:** Demographics and characteristics of the 505 consecutive papillary thyroid cancers retrospectively reviewed according to the presence or absence of chronic lymphocytic thyroiditis (CLT).

Parameters	CLT (*n* = 168)	non-CLT (*n* = 337)	*P*
Sex	146 (86.90%) F 22 (13.09%) M	253 (75.07%) F 84 (24.93%) M	0.002
F:M	6.64:1	3.01:1	–
Age (m ± SD)	44.42 ± 13.72	47.21 ± 13.76	0.03
Histotype	76 (45.23%) classic variant 65 (38.69%) follicular variant 16 (9.52%) sclerosing 3 (1.80%) tall cell 6 (3.57%) Warthin-like 2 (1.19%) hobnail/micropapillary 0 cribriform	180 (53.41%) classic variant 118 (35.01%) follicular variant 23 (6.82%) sclerosing 6 (1.78%) tall cell 3 (0.89%) Warthin-like 6 (1.78%) hobnail/micropapillary 1 cribriform (0.31%)	0.22
Lymph node	147 (87.5%) N0 21 (12.5%) N+	263 (78.04%) N0 74 (21.96%) N+	0.01
pT	110 (65.47%) T1a 38 (22.61%) T1b 12 (7.16%) T2 8 (4.76%) T3 0 T4	168 (49.85%) T1a 67 (19.88%) T1b 22 (6.52%) T2 79 (23.44%) T3 1 (0.31%) T4	<0.0001

**Figure 2:**
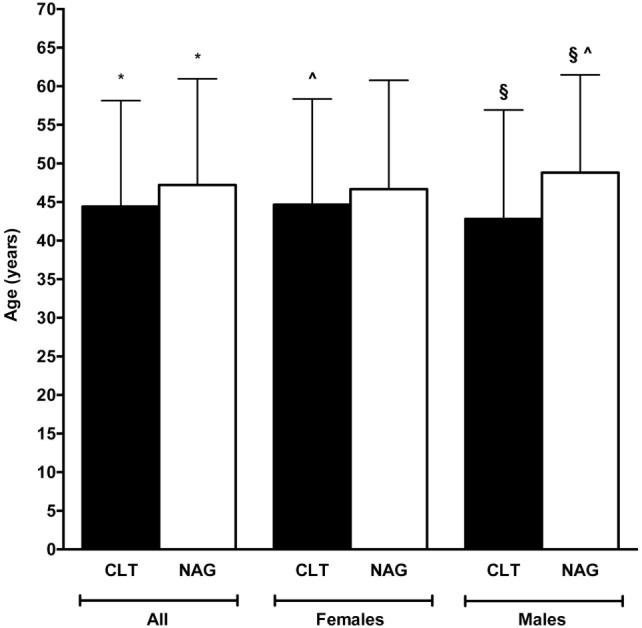
Comparison between patients’ age according to sex and presence (black bars) or absence (white bars) of chronic lymphocytic thyroiditis (CLT). Only significant (*P* < 0.05) or borderline significant (*P* between 0.05 and 0.10) statistical differences are reported. Symbols: *P = 0.03; ^§^*P* = 0.058; ^^^*P* = 0.02.

Age at diagnosis was not different between females and males (45.94 ± 13.96 vs. 47.55 ± 13.15 years, *P* = 0.272). Overall, CLT patients were younger compared with non-CLT patients (44.42 ± 13.72 vs. 47.21 ± 13.76 years, *P* = 0.03) (Figure 2). Stratifying by gender, this difference in age held, but only in males reached a borderline significance (42.82 ± 14.11 vs. 48.79 ± 12.68 years, *P* = 0.058). Non-CLT males were the eldest, and they were 4-year older compared with CLT females (48.79 ± 12.68 vs. 44.66 ± 13.69 years, *P* = 0.02) (Figure 2).

**Figure 3:**
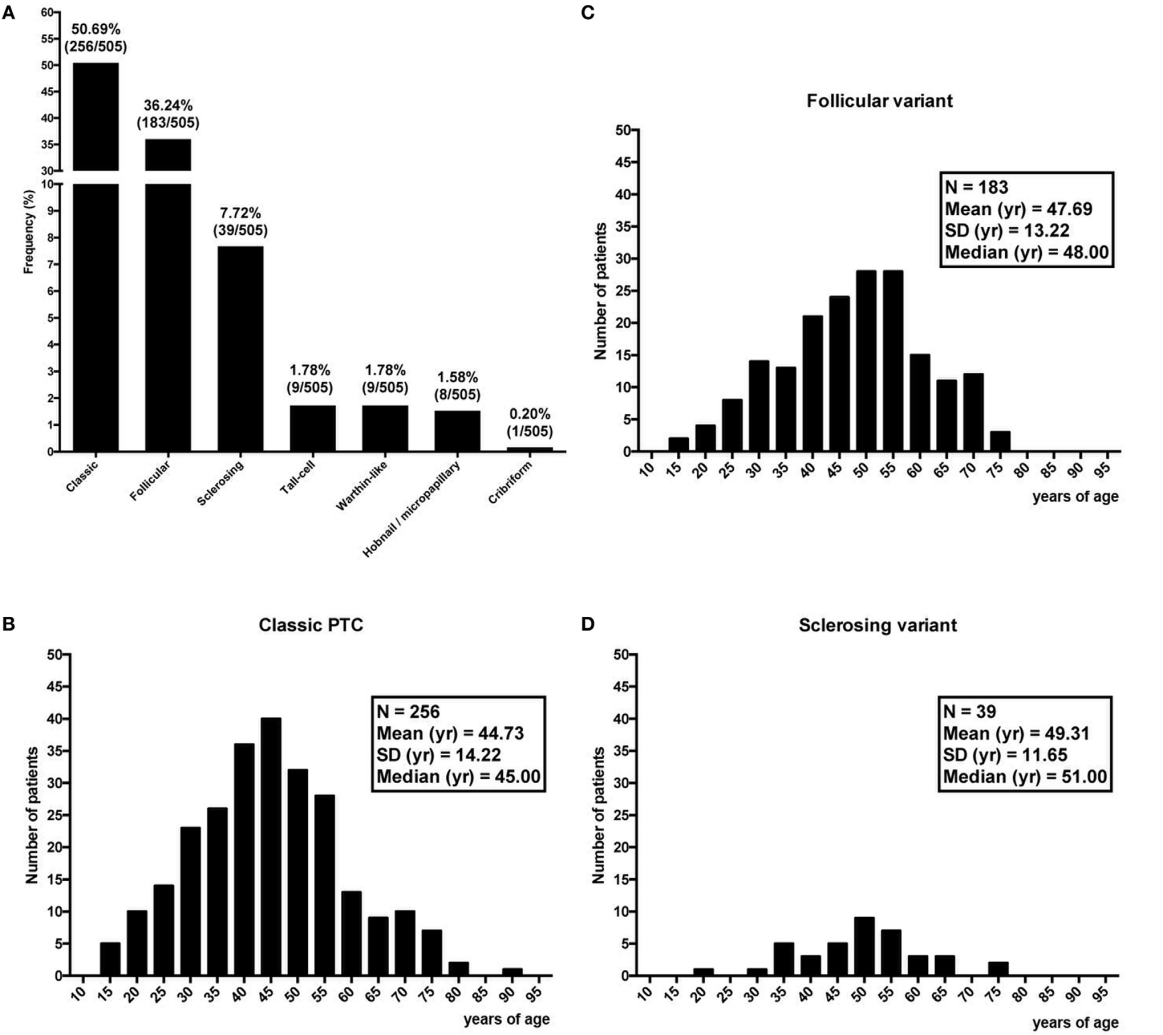

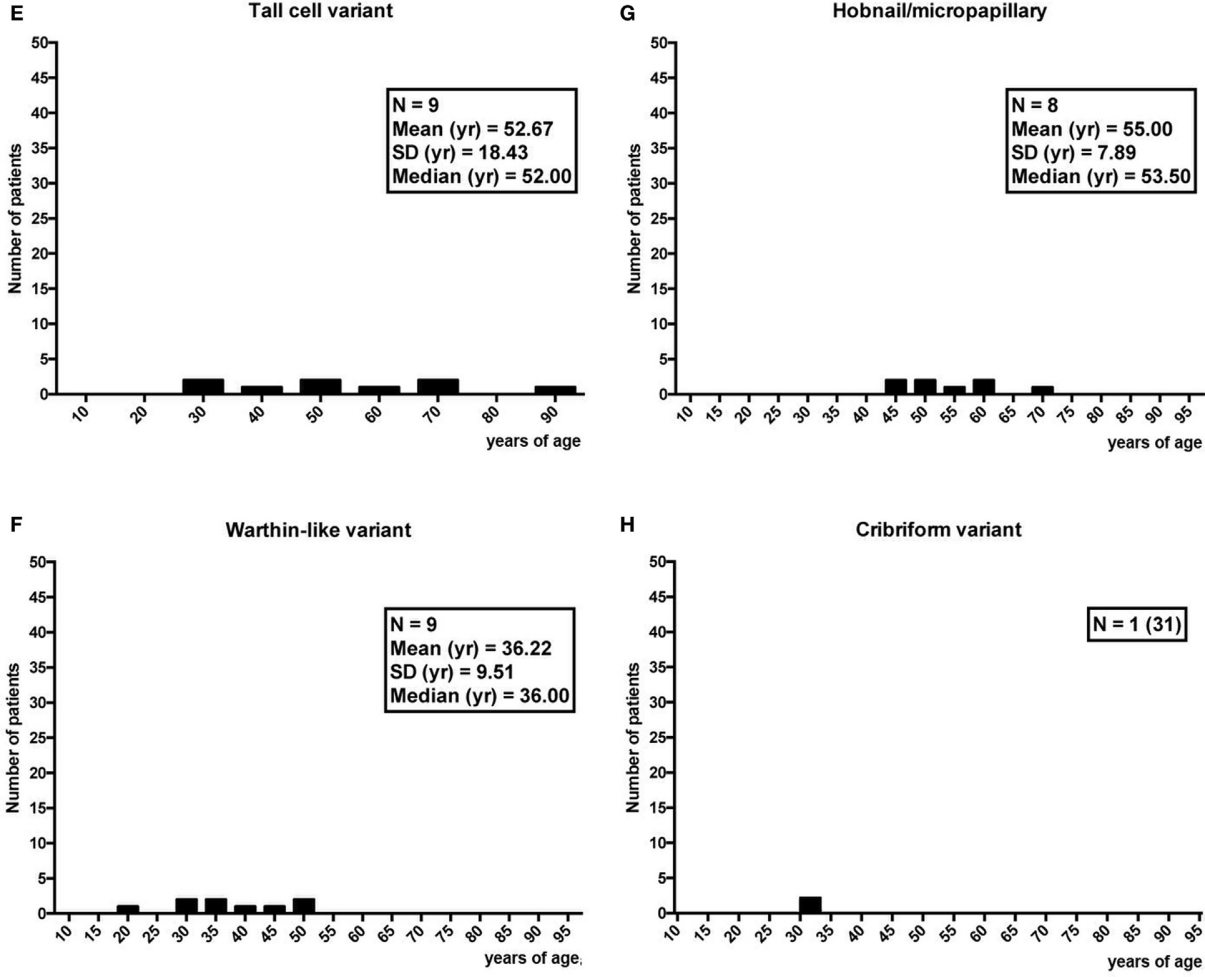
Frequency of the seven variants of papillary thyroid cancer **(A)**, and corresponding distribution of age **(B–H)**.

**Figure 4:**
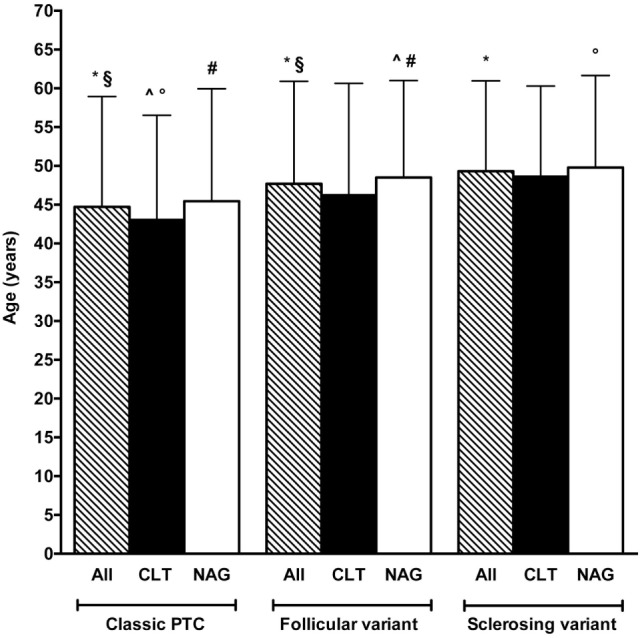
Comparison between age of patients according to histotype and presence (black bars) or absence (white bars) of chronic lymphocytic thyroiditis (CLT). The striped bars represent all patients. Note that only the three most frequent histotypes are reported (see Figure 3A). Only significant (*P* < 0.05) and borderline significant (*P* between 0.05 and 0.10) statistical differences are reported. Symbols: *P = 0.03; ^§^*P* = 0.03; ^^^*P* = 0.004; *P* = 0.03; ^#^*P* = 0.06.

Age varied greatly among histotypes (Figures 3A–H), with the lowest mean age in the Warthin-like group (36.22 years) (Figure 3F) and the highest in the hobnail/micropapillary group (55 years) (Figure 3G). Considering the three most frequent variants, namely the classic, the follicular, and the sclerosing (Figure 3A), the difference in age between CLT and non-CLT groups, with the first group being younger than the second group, held too (Figure 4). Moving across these three mentioned variants, age increased from the classic variant (44.73 ± 14.22 years) to the follicular variant (47.69 ± 13.22 years) and the sclerosing variant (49.31 ± 11.65 years) (*P* = 0.03) (Figure 4). Particularly, the youngest patients were those with the classic variant and histological findings of CLT, whereas the eldest patients were those with the sclerosing variant and without histological findings of CLT (43.03 ± 13.50 vs. 49.78 ± 11.88 years, *P* = 0.03). Concerning patients without signs of CLT, those with the classic variant were borderline significantly younger compared to those with the follicular variant (45.44 ± 14.49 vs. 48.50 ± 12.50 years, *P* = 0.06) (Figure 4).

**Figure 5:**
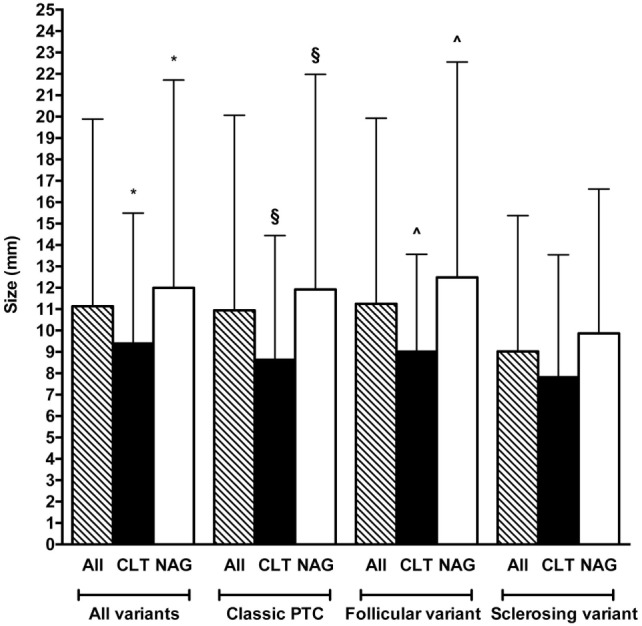
Comparison between tumor size according to histotype and presence (black bars) or absence (white bars) of chronic lymphocytic thyroiditis (CLT). The striped bars represent all patients. Note that only the three most frequent histotypes are reported (see Figure 3A). Only significant (*P* < 0.05) statistical differences are reported. Symbols: *P = 0.002; ^§^*P* = 0.008; ^^^*P* = 0.009.

Tumor size did not differ according to sex (data not shown) and histotype (Figure 5). Nevertheless, tumors with signs of CLT were smaller compared with the non-CLT ones (9.39 ± 6.10 vs. 12 ± 9.71 mm, *P* = 0.002). This difference in size was present in two of the three most prevalent variants, namely the classic and the follicular ones (*P* = 0.008 and *P* = 0.009, respectively) (Figure 5).

Lymph nodes were less frequently involved in CLT patients compared with non-CLT patients [21/168 (12.5%) vs. 74/337 (21.96%), *P* = 0.01, OR = 0.508, 95% CI = 0.30–0.86]. Concerning sex, this difference in lymph node involvement rate was present only in females (13.70 vs. 22.13%, *P* = 0.04, OR = 0.558, 95% CI = 0.32–0.97).

## Discussion

We retrospectively reviewed 505 consecutive PTC diagnosed over an 8-year time span. Exactly one-third (33.3%) of them had coexistent CLT. Compared to the patients CLT-negative, those with CLT were 3–6 years younger, particularly if they were females (Figure 2). Moreover, CLT patients had smaller tumors (Figure 5).

PTC is the most prevalent thyroid cancer, accounting for 70–80% of all thyroid malignancies (17). The association between thyroid cancer and inflammation was first described in the 1950s by Daley (18), but subsequent reports have been conflicting. For instance, a meta-analysis by Singh found an increased prevalence of CLT in PTC patients (19), whereas a systematic review including eight studies on fine-needle aspiration biopsy and nine studies on thyroid specimens, did not found any correlation between these two diseases (20). Yet, a meta-analysis on 38 studies showed a 23.2% rate of CLT in 10,648 PTC cases, with a preponderance of females and a protective role of CLT toward lymph nodes metastasis and extrathyroidal extension (7).

Recently, a systematic review and meta-analysis analyzing 36 studies (8) found an association between CLT and PTC or thyroid lymphoma, but not between CLT and follicular, medullary, or anaplastic cancer. Particularly, one-fifth (18.9%) of PTC cases had CLT, in contrast with the 33.3% rate found in the present study, a twofold difference. This difference may be explained by the heterogeneity of the studied analyzed by Resende de Pavia et al., which included different diagnostic criteria of CLT (biochemical, sonographic, cytological, and histological) (8). An additional explanation can be geographical in nature. In our geographical area, the incidence of autoimmune thyroiditis has increased over 10-fold compared to the 1970s and 1990s, with age at presentation dropped by almost one decade (2, 4).

A number of studies have shown that PTC patients with CLT have a better prognosis (21–23). Indeed, CLT patients undergo thyroid ultrasound more frequently compared to patients without CLT, with greater chance to detect small suspicious thyroid nodules. Not unexpectedly, in the present study, the average size of PTC in CLT patients was lower than 1 cm (9.39 mm), which was 3 mm smaller than the average size in the non-CLT patients (Figure 5).

The relationship between thyroid cancer and autoimmune thyroiditis is tight, but its mechanistic occurrence is far to be elucidated. Whether the first is the consequence of the second or *vice versa* is still debated. Evidence is that chronic inflammation might act as a trigger for neoplastic transformation. In turn, neoplastic cells are capable to produce inflammatory cytokines. Our data are in accordance with the previous studies (8, 23), showing that thyroid cancer is diagnosed earlier in CLT patients compared with those without autoimmune thyroiditis, probably because they undergo periodically thyroid ultrasound. As a result, microcarcinomas and macrocarcinomas as well are diagnosed more frequently at an earlier stage in patients with CLT compared to patients without CLT. Accordingly, CLT patients would be in the favorable position of having smaller tumors that are detected at an earlier stage.

In conclusion, this study from the northeast Sicily reports a high rate of CLT in PTC, and a TNM staging that is in line with the favorable impact of autoimmune thyroiditis on prognosis of PTC.

## Author Contributions

AI, GT, and SB conceived the study. AI, EM, MS, and GT reviewed the records and collected the data. AI, RV, FB, SB, and GT analyzed the data. RV performed statistics. AI, RV, GT, and SB drafted the manuscript. All authors contributed to the final revision of the manuscript.

## Conflict of Interest Statement

The authors declare that the research was conducted in the absence of any commercial or financial relationships that could be construed as a potential conflict of interest.

## Abbreviations

CLT, chronic lymphocytic thyroiditis; PTC, papillary thyroid cancer.
